# The relationship between effort-reward imbalance and presenteeism among nurses: a cross-sectional study from China

**DOI:** 10.3389/fpubh.2026.1764086

**Published:** 2026-04-08

**Authors:** Zehua Li, Zongting Luo, Yanrui Tan, Yue Chang, Xiaolin Ma

**Affiliations:** The Third People's Hospital of Chengdu, Chengdu, Sichuan, China

**Keywords:** cross-sectional study, effort-reward imbalance, health, nurse, presenteeism

## Abstract

**Background:**

Currently, nurses are under great pressure and prone to the effort-reward imbalance and presenteeism, which hinders the development of medical health and has gained insufficient attention. The objective of this study is to investigate the actual situation of the effort-reward imbalance and presenteeism for nurses, alongside the exploration of the relationships between them, which is aimed to provide guidance for the alleviation of presenteeism and improvement of nursing quality.

**Methods:**

This study was a cross-sectional survey. From October to December 2024, a convenience sampling method was used to conduct a survey among 2,095 nurses from four hospitals in Sichuan Province, China. Data were collected through Questionnaire Star. The questionnaire was composed of general data, the effort-reward imbalance scale and the Stanford Presenteeism Scale. Data analysis was conducted using SPSS 26.0 statistical software, including descriptive statistics and Pearson correlation analysis.

**Results:**

The effort-reward imbalance score of nurses was (55.91 ± 8.32), in which 52.1% of nurses were in the effort-reward imbalance state, and 44.9% of nurses were in the high-overcommitment state. The presenteeism score of nurses was (16.82 ± 5.85). Besides, the presenteeism of nurses under the state of effort-reward imbalance was relatively higher than those with effort-reward balance (*t* = −43.30, *P* < 0.001), and the presenteeism of nurses with high-overcommitment was higher than others (*t* = −41.77, *P* < 0.001). Overall, effort-reward imbalance and presenteeism were positively correlated (*r* = 0.340, *P* < 0.001).

**Conclusion:**

The condition of effort-reward imbalance is serious among Chinese nurses, alongside the common presenteeism. Besides, effort-reward imbalance and presenteeism were positively correlated. Overall, it is recommended to alleviate the workload of nurses appropriately, alongside the improvement of their rewards and more attention to their physical and mental health.

## Introduction

With the continuous promotion of the Healthy China strategy in recent years, the quality of medical services has been continuously improved in medical institutions, alongside the sustained protection of the people's health. However, with the rapid development of society and the increasing service demand, the physical and mental health problems faced by nurses require more attention. Nurses serve as the main group in the medical system, which are the main factors in the development of high-quality nursing in hospitals (1). However, their physical and mental health is greatly challenged, which could be attributed to the particularity of the profession and the great pressure from work. Additionally, nurses tend to work in a complex environment, which often exposes them to occupational hazards, such as physical injury, contact with infectious or toxic substances, and allocation of dangerous drugs related to chemotherapy (2, 3). In addition, nursing work is characterized by tedious work, high load, night shift, multiple tasks, high pressure and low substitutability (4, 5). At present, nurses are at a relatively low status in China, alongside the low income and insufficient nursing resources (6, 7). Overall, the above dilemmas result in severe challenges to the physical and mental health of nurses, simultaneously decreasing their work efficiency (8). Therefore, it is in urgent need to develop scientific guidance for nurses' psychological problems, alongside more attention to their physical and mental health, in order to guarantee their work efficiency and improve nursing quality.

Presenteeism refers to the phenomenon that individuals insist on working when they exhibit physical and mental health problems. Besides, their working enthusiasm shows a decreasing trend and they cannot devote themselves to work, resulting in low work efficiency and decreased productivity (9, 10). It was revealed by studies that nurses exhibited a high incidence of presenteeism due to the problems of high-intensity, high-load and high-risk work (11, 12), and the incidence of presenteeism was 3–4 times that of other industries (13). Additionally, the impact of presenteeism on individuals and organizations is much higher than that of absenteeism (14). Moreover, the body and mind of nurses could not fully recover from presenteeism, ultimately leading to reduced work efficiency and job burnout (15). In addition, presenteeism tends to result in the loss of hospital productivity, which would directly or indirectly induce economic losses (16). Therefore, it is required to pay more attention to nurses' presenteeism.

The effort-reward imbalance was proposed by Siegrist, and it was mainly employed in the evaluation of the social psychological factors of individuals in the work environment, which was mainly composed of the imbalance in working hours, salary, working pressure, promotion opportunities, job prospects, respect and other aspects (17). As a work stress theory, the effort-reward imbalance model refers to the mismatch between high efforts and low rewards, which can be used in the prediction of physical and mental health problems of individuals (18). It has been revealed by relevant studies that most nurses are in a state of effort-reward imbalance due to the particularity of work, accounting for up to 64.7% (19). Notably, the effort-reward imbalance tends to result in physical and mental health problems, such as migraines, coronary heart disease, job burnout and depression among nurses (20–22). Therefore, it is of great significance to pay attention to the effort-reward imbalance of nurses.

The physical and mental health of medical workers has become a hot spot of research by scholars for a long time. At present, the correlation between effort-reward imbalance, job burnout and turnover intention is the predominant focus of scholars (23, 24). However, empirical evidence regarding the effort-reward imbalance and presenteeism among Chinese nurses is still relatively scarce. Moreover, most of the existing studies adopt small sample designs and lack validation with large samples. Nurses serve as the main force of health human resources, which are on the front line of medical and health care, alongside the closest contact with patients. Moreover, nurses tend to exhibit crucial functions in the improvement of patients' health and promoting medical development. However, nurses are more prone to physical and mental health problems under a heavy burden, which is more likely to lead to the effort-reward imbalance and presenteeism. Based on this background, the effort-reward imbalance and presenteeism of nurses are investigated in this study based on the model of effort-reward imbalance, alongside the discussion of the impact of effort-reward imbalance on presenteeism, which is aimed to provide a reference for the early identification of presenteeism in nurses and improvement of physical and mental health.

## Method

### Study design and samples

This study conducted an online questionnaire survey among clinical nurses in 4 hospitals in Sichuan Province, China, from October to December 2024, with the application of convenience sampling. A total of 2,141 questionnaires were collected in this study. After excluding 46 invalid questionnaires, 2,095 valid questionnaires were obtained, with an effective rate of 97%.

Inclusion criteria: possess a nurse professional qualification certificate; Informed consent and voluntary participation in the study.

Exclusion criteria: absentees due to personal leave, maternity leave, etc.; A nurse who is in practice (student nurses), advanced study or other training.

According to Kendall's sample size calculation method, the sample size is at least 5–10 times the number of independent variables (25). Additionally, this study includes 16 variables, and the sample size ranges from 96 to 192 with the consideration of a 20% rate of invalid questionnaires. Therefore, the sample size of this study is sufficient.

### Measures

#### General information questionnaire

The questionnaire was designed by the researcher according to the literature review and research group discussion. Besides, the questionnaire was mainly composed of 11 variables, such as age and gender.

#### The effort-reward imbalance scale

The effort-reward imbalance scale was compiled by Siegrist (17) and then translated by Chinese scholars (26). 23 items were present in this scale, which could be divided into three aspects: effort (six items), reward (11 items), and Over Commitment (six items). Besides, a Likert four-point rating was applied, ranging from 1 (strongly disagree) to 4 (strongly agree), with some entries scored in reverse. The ERIratio is employed in the assessment of the imbalance between effort and reward. Effort-reward ratio was calculated as follows: (ERIratio) = Effort/(reward × 6/11), in which ERIratio > 1 indicated that effort-reward was in an unbalanced state, while ERIratio ≤ 1 could indicate that effort-reward was relatively balanced. In the overcommitment dimension, the limit is defined by the top 1/3 of the scores of overcommitment items as the high-overcommitment state (the overcommitment dimension score is 18 points), and score ≥ 18 was classified as the high-overcommitment state. Additionally, the Cronbach's α coefficient of this scale was 0.885.

#### The presenteeism scale

The Stanford Presenteeism Scale-6 (SPS-6) was employed in the assessment of presenteeism. This scale was developed by Koopman et al. (27) and translated into Chinese by Zhao et al. (28), which consisted of six items across two dimensions. Besides, a five-point Likert scale was used, ranging from 1 (completely disagree) to 5 (completely agree), in which items 5 and 6 were scored in reverse. The total score ranged from 6 to 30, in which higher scores indicated more severe presenteeism. Additionally, the Cronbach's α coefficient of this scale was 0.972.

### Ethical consideration

This study was approved by the Ethics Committee of the Third People's Hospital of Chengdu [(2024)-S-263]. Data collection was conducted in agreement with the principles of anonymity and informed consent.

### Procedures

Questionnaire Star electronic questionnaire was employed to collect the data. The research team contacted the nursing directors of each hospital via phone and WeChat, explained the research purpose and obtained their consent. Then, the nursing department forwarded the questionnaire link to the clinical nurses' work WeChat group through the department nurse leaders. The first page of the questionnaire clearly stated the research purpose, the principle of voluntary participation and confidentiality measures, and set a rule that each IP address and WeChat account could only be filled out once. After the investigation, the collected data were sorted out, and the questionnaires with inconsistent logic and a high repetition rate were eliminated.

### Statistical analysis

SPSS 26.0 statistical software (IBM Corporation, Armonk, NY, USA) was used for the data analysis in this study. The measurement data of the normal distribution was represented as $\bar{x}$ ± *S*, and the counting data was described by frequency and percentage. Additionally, one-way ANOVA or independent sample *t*-test was used for inter-group analysis, and Pearson correlation analysis was used for inter-group correlation analysis. Univariate variables with *P* < 0.05 were included in the regression model, followed by the implementation of the multivariate regression analysis.

## Results

### General data of nurses

According to the results, the 2,095 nurses in this study were 20–55 (32.66 ± 7.17) years old with 1–38 (10.82 ± 7.70) years of work, and the detailed information was listed in Table 1.

**Table 1:** Analysis of presenteeism among nurses with different demographic characteristics and effort-reward imbalance (*n* = 2,095).

Item		Number of cases (%)	Total score of presenteeism	*F/t* value	*P*-value
Department	Internal medicine	675 (32.2)	17.04 ± 5.79	3.315	0.010
	Surgery	451 (21.5)	17.20 ± 5.76		
	Obstetrics and gynecology	206 (9.8)	15.99 ± 6.08		
	Operating room and others	626 (29.9)	16.41 ± 5.95		
	Emergency and severe disease	137 (6.5)	17.69 ± 5.37		
Gender	Male	105 (5.0)	17.88 ± 5.27	1.891	0.059
	Female	1,990 (95.0)	16.77 ± 5.87		
Age (years)	≤ 30	964 (46.0)	17.58 ± 5.63	16.279	< 0.001
	31–40	826 (39.4)	16.35 ± 5.94		
	≥41	305 (14.6)	15.74 ± 5.98		
Education	Junior college	816 (38.9)	17.33 ± 5.79	3.141	0.002
	Bachelor degree or above	1,279 (61.1)	16.50 ± 5.86		
Years of work	≤ 5	601 (28.7)	17.53 ± 5.39	14.370	< 0.001
	6–10	596 (28.4)	17.28 ± 5.99		
	≥11	898 (42.9)	16.05 ± 5.96		
Marital status	Married	1,471 (70.2)	16.62 ± 5.93	4.341	0.013
	Unmarried	568 (27.1)	17.43 ± 5.54		
	Divorced/widowed	56 (2.7)	16.11 ± 6.24		
Professional post	Junior	1,160 (55.4)	17.28 ± 5.79	8.508	< 0.001
	Middle level	822 (39.2)	16.18 ± 5.86		
	High-level	113 (5.4)	16.81 ± 5.98		
Average monthly income (RMB)	< 5,000	898 (42.9)	17.63 ± 5.61	15.660	< 0.001
	5,000–10,000	1,144 (54.6)	16.26 ± 5.95		
	>10,000	53 (2.5)	15.38 ± 6.02		
Average monthly night shifts (times)	< 4	994 (47.4)	16.27 ± 6.00	14.899	< 0.001
	4–8	789 (37.7)	16.93 ± 5.65		
	>8	312 (14.9)	18.32 ± 5.60		
Average overtime h per week (h)	< 5	1,487 (71.0)	16.40 ± 5.87	14.065	< 0.001
	5–10	463 (22.1)	17.75 ± 5.61		
	>10	145 (6.9)	18.23 ± 5.79		
Nature of employment	Contract employment or otherwise	1,837 (87.7)	16.90 ± 5.80	1.508	0.132
	Officially on the list	258 (12.3)	16.31 ± 6.14		
Overcommitment	< 18	1,155 (55.1)	13.27 ± 5.07	−41.775	< 0.001
	≥18	940 (44.9)	21.20 ± 3.16		
ERI	≤ 1	1,003 (47.9)	12.63 ± 5.13	−43.309	< 0.001
	>1	1,092 (52.1)	20.68 ± 3.23		

### Effort-reward imbalance and presenteeism of nurses

The nurses' effort-reward imbalance score in this study was (55.91 ± 8.32) points, and the presenteeism score was (16.82 ± 5.85) points, as detailed in Table 2. Additionally, 44.9% of nurses were in the state of high-overcommitment according to the classification of overcommitment and ERI, while 52.1% of nurses were in the state of effort-reward imbalance.

**Table 2:** Effort-reward imbalance and presenteeism score of nurses (*n* = 2,095).

Item	Possible range	Observed range	Score (score, $\bar{x}$ ±*S*)
Effort-reward imbalance	23–92	34–76	55.91 ± 8.32
Effort	6–24	6–24	15.51 ± 4.18
Reward	11–44	11–44	24.76 ± 5.76
Overcommitment	6–24	6–24	15.63 ± 3.83
Presenteeism	6–30	6–30	16.82 ± 5.85
Work limitation	4–20	4–20	11.34 ± 4.24
Work energy	2–10	2–10	5.49 ± 1.88

### Analysis of nurses' presenteeism with different demographic characteristics and effort-reward imbalance

As shown in Table 1, the differences among different departments, age, education, working years, marital status, professional title, income, night shift frequency, overtime hours, overcommitment and ERI presenteeism were statistically significant (*P* < 0.05). Among them, the presenteeism of nurses in the Emergency and severe disease departments, aged ≤ 30 years old, years of work ≤ 5 years, unmarried, junior professional post, average monthly income < 5,000 RMB, average monthly night shifts >8 times, and average overtime hours per week >10 h were significantly higher vs. those of other groups.

### Correlation analysis between nurses' effort-reward imbalance and presenteeism

The nurses' effort-reward imbalance score in this study was positively associated with presenteeism score (*r* = 0.340, *P* < 0.001), and the correlation of scores in each dimension was shown in Table 3.

**Table 3:** Correlation analysis between nurses' effort-reward imbalance and presenteeism (*n* = 2,095).

Item	Effort	Reward	Overcommitment	Effort-reward imbalance	Work limitation	Work energy	Presenteeism
Effort	1	—	—	—	—	—	—
Reward	−0.289^**^	1	—	—	—	—	—
Overcommitment	0.880^**^	−0.236^**^	1	—	—	—	—
Effort-reward imbalance	0.708^**^	0.438^**^	0.740^**^	1	—	—	—
Work limitation	0.680^**^	−0.431^**^	0.650^**^	0.343^**^	1	—	—
Work energy	0.626^**^	−0.435^**^	0.585^**^	0.283^**^	0.794^**^	1	—
Presenteeism	0.695^**^	−0.453^**^	0.659^**^	0.340^**^	0.981^**^	0.898^**^	1

^**^All *P* < 0.001.

### Regression analysis of the influence of effort-reward imbalance on presenteeism

The total score of the nurses' presenteeism scale was employed as the dependent variable in regression analysis, while the variables with statistical significance in the general data and the score of effort-reward imbalance were used as the independent variables. It could be revealed that education, income, night shifts, overtime hours and effort-reward imbalance served as the influencing factors of nurses' presenteeism (*P* < 0.05), as shown in Table 4.

**Table 4:** Regression analysis of the influence of nurses' effort-reward imbalance on presenteeism (*n* = 2,095).

Item	*B*	*SE*	β	*t*	*P*-value	***95% CI***		VIF
						Lower limit	Upper limit	
Constant	5.648	1.663	—	3.397	0.001	2.388	8.909	—
Education	−0.680	0.270	−0.057	−2.521	0.012	−1.209	−0.151	1.235
Income	−0.724	0.251	−0.067	−2.881	0.004	−1.216	−0.231	1.310
Night shift	0.445	0.186	0.055	2.392	0.017	0.080	0.809	1.274
Overtime hours	0.767	0.201	0.080	3.823	< 0.001	0.374	1.160	1.058
Effort-reward imbalance	0.227	0.015	0.323	15.594	< 0.001	0.199	0.256	1.049

*R* = 0.384; *R*^2^ = 0.148; adjusted *R*^2^ = 0.142; *F* = 25.711; *P* < 0.001.

## Discussion

### The presenteeism is common among Chinese nurses

It was revealed that the presenteeism score of nurses was (16.82 ± 5.85). This finding is consistent with the high prevalence of presenteeism among nurses worldwide. For instance, the presenteeism rate among nurses in the Netherlands reached 50%, and 52.6% of American nurses reported experiencing presenteeism in the past 4 weeks (29, 30). Nursing served as an important part within the medical and health field, and nurses tended to exhibit crucial functions in ensuring the safety of patients, alongside the promotion of the recovery and prognosis of patients. Besides, nurses were prone to presenteeism and other physical and mental health problems, which could be attributed to their irregular working hours, tense working environment, heavy workload, high work pressure and long-term stress (31). Additionally, presenteeism of nurses exhibited a strong connection with job burnout and occupational stress, leading to adverse nursing events and additional economic losses of patients and hospitals suffer, which was an important factor for patient safety, nursing quality and medical cost (32, 33). Overall, it was in urgent need for hospital administrators and society to pay more attention to the presenteeism of clinical nurses, alongside the implementation of corresponding improvement measures (34). It was recommended to focus on the physical and mental condition of nurses, advocacy of working without illness, enhancement of the construction of the nursing team, which was aimed to ensure the best attendance of nurses, avoid adverse events, and ensure the safety of patients. For instance, establish a mechanism for screening nurses' mental health, use standardized tools (such as the PHQ-9 depression scale) for assessment, and provide psychological counseling services. In addition, it was suggested to pay more attention to clinical nurses with short working years, low professional titles, more night shifts and low income, scientific scheduling and post setting, reasonable arrangement of work content and timely length, which was aimed to reduce the workload and intensity of nurses, alongside the improvement of the working environment and reduction of the presenteeism incidence. Given the cross-sectional design of this study, the above recommendations should be regarded as exploratory and hypothesis-generating rather than validated intervention measures.

### Effort-reward imbalance is prevalent among Chinese nurses

In this study, 52.1% of the nurses were in an effort-reward imbalance state, which is highly consistent with the 52.3% global average reported in a recent international meta-analysis (35). This might be related to the shortage of nurses' human resources and the special characteristics of nurses' work. According to a report by the World Health Organization, there were 27.9 million nursing practitioners worldwide. Currently, the total number of registered nurses in China has exceeded 5.2 million, but the nurse human resources are still lacking (36). In addition, nursing work was inherently characterized by high work intensity, frequent night shifts, long and irregular working hours, and significant psychological pressure. However, most nurses in China were contract nurses, and their salaries and job stability were relatively poor. Meanwhile, compared with the doctors, Chinese nurses received far lower salaries, social status, respect, promotion opportunities, etc. (37). With the continuous progress of society, residents' health consciousness exhibited an increasing trend, which resulted in a high demand for nursing services, which resulted in an increase in nurses' workload and pressure, alongside the aggravation of the effort-reward imbalance of nurses (38). Besides, the presenteeism score of nurses in the effort-reward imbalance state was higher than that of nurses in the effort-reward balance state, and the presenteeism scores of nurses with high-overcommitment were also higher than others. It could be indicated that long-term high load and low return exhibited a negative impact on the physiology, psychology and behavior of nurses, inducing damage to their physical and mental health. Overall, after heavy work without any feedback on salary, professional title promotion, welfare benefits, professional title promotion and career development, nurses were likely to have negative psychology such as dissatisfaction, boredom and burnout, and even presenteeism (39). It is suggested to establish a transparent performance evaluation mechanism and a clear career development path, ensuring that salary increases and professional title promotions fairly reflect the labor value of nurses. A specialized nurse certification system should be established, along with opportunities for continuing education (such as master's or doctoral studies) and participation in departmental decision-making, to enhance the organizational identification of nurses. These suggestions are exploratory hypotheses based on research and need to be verified through future intervention studies.

### The effort-reward imbalance of nurses is positively correlated with presenteeism

It was revealed that effort-reward imbalance and presenteeism were positively correlated among nurses. It was suggested that effort-reward imbalance among nurses showed an impact on their presenteeism. Moreover, this correlation may be mediated by suboptimal health status (SHS), a transitional pre-disease state marked by fatigue, distress, and reduced vitality. ERI strongly associates with elevated SHS in nurses (OR = 4.35, 95% CI: 3.66–5.17) (40), potentially leading to presenteeism as impaired health prompts continued work despite lower efficiency. Targeting SHS early could disrupt this pathway; future studies should test mediation effects. Therefore, it was important to pay more attention to the issue of effort-reward imbalance among nurses, which was aimed to reduce the occurrence of presenteeism. According to the effort-reward imbalance model, effort was mainly composed of working time, work burden, etc., while rewards were mainly composed of salary, promotion opportunity, job prospect, respect, etc. Based on this theoretical framework, we have put forward the following management suggestions that need to be verified. The work responsibilities and value of nurses required to be further clarified by hospital managers, in order to create a positive working atmosphere, and improve the mental health of nurses. Additionally, managers should make reasonable scheduling and division of labor and optimize the work structure and process, in order to reduce the workload and pressure on nurses. Simultaneously, the human resources of nurses should be reasonably regulated to reduce the high-load and high-intensity work caused by the shortage of human resources (41). For instance, in accordance with the standard ratio of nurses to patients, additional nurses should be deployed in departments such as emergency rooms, operating rooms, and intensive care units. Secondly, it was required to establish a rational performance evaluation and promotion mechanism, alongside the increase of the salary and benefits of nurses, which could construct a reward mechanism for their labor value. Besides, managers should also increase their organizational identity and career development opportunities, such as providing nurses with the opportunity to pursue higher education, further training, and participation in significant departmental decision-making, etc., which could contribute to the sense of work control, recognition and respect, enhancement of professional identity, and reduction of the effort-reward imbalance (42). Furthermore, it is suggested that future intervention studies focus on the mental health of clinical nurses. In addition to early prevention, detection, and intervention of mental health issues, efforts should be made to explore the effectiveness of providing psychological counseling and guidance to nurses. For instance, introducing mindfulness-based stress reduction programs and resilience training.

However, there were some limitations in this study: Firstly, in this study, convenience sampling was only adopted for nurses in Sichuan Province, China. It may only represent a specific type of medical institution or nurse group (as this study mainly focused on female nurses and the proportion of male nurses was too low), and it cannot reflect the entire population of nurses in China, resulting in sampling bias. Therefore, future research should expand the geographical scope to multiple centers across the country and adopt stratified random sampling methods, conducting investigations based on hospital grade, department type, gender, age, etc., in order to ensure the representativeness of the sample and reduce selection bias. Secondly, this study is a cross-sectional study. It not only fails to determine the causal relationship between variables, but also may have potential reverse causal relationships. That is, not only can the effort-reward imbalance lead to presenteeism, but presenteeism may also reversely exacerbate the effort-reward imbalance. Unmeasured residual confounding factors such as organizational atmosphere may also affect the relationship between variables. Future research can adopt longitudinal designs to clarify the causal relationship and systematically measure potential confounding factors. Finally, this study is mainly dependent on self-reported measurements, in which participants may introduce biases due to various factors, such as overestimating or underestimating their own behaviors and experiences. These deviations may have an impact on the validity of the research conclusions, such as the exaggeration and underestimation of the strength of the association between variables. Overall, the combination of novel data collection methods could be employed in future research, such as the observation method, which could reduce the bias introduced by self-reporting.

## Conclusions

Overall, it was found that the phenomenon of presenteeism was relatively high among nurses, alongside the common situations of effort-reward imbalance and high-overcommitment. Therefore, relevant health departments should pay more attention to presenteeism and the effort-reward imbalance of nurses, alongside the scientific channel of nurses' psychological problems. Moreover, it is necessary to focus on their physical and mental health, alongside the reasonable adjustment of work requirements and reduction in redundant work. Additionally, the rewards of nurses receiving from their work should comprehensively consider the aspects of salary, career promotion, respect, etc., in order to avoid the imbalance between effort and rewards.

## Data Availability

The raw data supporting the conclusions of this article will be made available by the authors, without undue reservation.
